# DM-MQTT: An Efficient MQTT Based on SDN Multicast for Massive IoT Communications

**DOI:** 10.3390/s18093071

**Published:** 2018-09-12

**Authors:** Jun-Hong Park, Hyeong-Su Kim, Won-Tae Kim

**Affiliations:** The Department of Computer Science and Engineering, Korea University of Technology and Education, Cheonan-si 31253, Korea; astrada1@koreatech.ac.kr (J.-H.P.); there9@koreatech.ac.kr (H.-S.K.)

**Keywords:** MQTT, low delay, multicast, SDN, edge computing

## Abstract

Edge computing is proposed to solve the problem of centralized cloud computing caused by a large number of IoT (Internet of Things) devices. The IoT protocols need to be modified according to the edge computing paradigm, where the edge computing devices for analyzing IoT data are distributed to the edge networks. The MQTT (Message Queuing Telemetry Transport) protocol, as a data distribution protocol widely adopted in many international IoT standards, is suitable for cloud computing because it uses a centralized broker to effectively collect and transmit data. However, the standard MQTT may suffer from serious traffic congestion problem on the broker, causing long transfer delays if there are massive IoT devices connected to the broker. In addition, the big data exchange between the IoT devices and the broker decreases network capability of the edge networks. The authors in this paper propose a novel MQTT with a multicast mechanism to minimize data transfer delay and network usage for the massive IoT communications. The proposed MQTT reduces data transfer delays by establishing bidirectional SDN (Software Defined Networking) multicast trees between the publishers and the subscribers by means of bypassing the centralized broker. As a result, it can reduce transmission delay by 65% and network usage by 58% compared with the standard MQTT.

## 1. Introduction

Cloud computing shows problems when many IoT (Internet of Things) devices send and receive data like smart cities. Existing cloud computing collects and analyzes data generated from multiple devices in a single cloud. As the number of IoT devices grows, the burden of processing data in a single cloud increases [1,2,3]. The evolution of IoT devices enables the transfer of large amounts of data, such as photographs and video data, rather than small sensor data, resulting in network congestion in the process of collecting data in the cloud [4,5,6]. Since IoT devices evolve from devices that produce data to devices that produce and consume data, sending data to the cloud for analysis and returning the analyzed results to the IoT device is an unnecessary delay. Therefore, to improve the problem of cloud computing, recent research suggests edge computing [7,8,9].

Edge computing and cloud computing are different in the process of collecting data for analysis. Edge computing, like cloud computing, requires the transfer of data collected from IoT devices to the server for analysis. An edge computing analysis server, called an edge node, is placed close to the IoT device that generates and consumes the data [10]. The cloud server of edge computing selects only the data that needs to be stored, and collects it at the edge node, which can reduce the bottleneck due to network concentration. In addition, edge nodes are built close to IoT devices, and edge computing reduces unnecessary transfer latency compared to cloud computing [11]. Therefore, edge computing with massive IoT devices should efficiently use computing resources for data analysis.

IoT communication middlewares, used for IoT data transmission, are also required to be changed, according to the edge computing paradigm. The MQTT (Message Queuing Telemetry Transport) protocol is a broker-based IoT middleware that is widely used in IoT systems [12]. The location of the broker is important because the MQTT protocol exchanges data through topics, and all devices with the same topic must be connected to the broker [13]. When a broker is installed on all edge nodes to use the MQTT protocol in edge computing, the complexity of the connection increases, and the problem of managing a large number of brokers arises. The IoT device should know the location of the broker with the topic, so that an IoT device belonging to a particular edge network subscribes to the topic of another edge network. Since IoT devices send a persistent message to maintain connectivity with the broker, this process can cause network congestion due to multiple brokers installed on the edge node. Therefore, a mechanism is needed to manage brokers installed on all edge nodes, and to exchange data between multiple edge networks without additional connection to brokers.

An additional mechanism is required for the MQTT protocol to transfer data between multiple edge networks. When devices in multiple edge networks subscribe to a specific topic in a particular edge network, congestion of the network increases if data is transmitted in unicast. Even with the broker-less mechanism, scalability is reduced, due to the complex communication links between multiple IoT devices. The standard MQTT protocol performs multicast operations because it sends data from the broker to multiple subscribers based on unicasting, but does not use the actual multicast routing. In massive IoT communications, delays in the broker can have a significant impact on the speed of the system if there are a large number of subscribers. In order to add multicast to the existing MQTT, multicast groups should be managed by classifying the topics of devices existing in a plurality of edge networks. In addition, it is necessary to manage data classified into QoS (Quality of Service) level. The QoS of the MQTT protocol is divided into three levels [14]. Since the sender and the receiver must be set to the same level, group management according to the QoS level is required.

Emergency data transport between multiple edge networks is a consideration in the edge network. Since the edge network is installed close to the device and is composed of the small area, it is difficult for the edge network to have an emergency response server in every edge network. When a disaster situation occurs on a particular edge network, it is necessary to send emergency data to the emergency response servers present on the other edge network. Improving the MQTT protocol to reduce network congestion and reserve bandwidth for emergency data is effective for rapid transmission of emergency data. 

The authors in this paper propose a mechanism that manages all pub/sub devices by hierarchically configuring the brokers of the cloud and the edge nodes. SDN (Software Defined Networking) and DM-MQTT (Direct Multicast-MQTT) minimize data transfer delays between multiple edge networks [15]. The master broker in the cloud collects the IP addresses, QoS level, and topics of the pub/sub devices connected to the slave broker from all edge nodes and sends them to the SDN controller. The SDN controller uses the information transmitted from the master broker to create a group in consideration of the topic, the QoS level, and the emergency data, and then sets a data transmission path. DM-MQTT with multicast minimizes data transfer delays between pub/sub devices in distributed edge networks.

## 2. Related Works

Many types of IoT middleware have been studied to connect devices and transmit data in massive IoT systems. The functional comparison between IoT middleware is shown in Table 1.

DDS (Data Distribution Service) is a middleware defined by OMG (Object Management Group) that supports 22 QoS policies and multicast [16,17]. Compared to other IoT protocols, DDS, which is a relatively heavyweight protocol, has a weakness that it is difficult to apply to low-power devices with low computing power. CoAP (Constrained Application Protocol) is a standardized protocol in the IETF (Internet Engineering Task Force) CoRE (Constrained RESTful Environments) working group to exchange data using the RESTful method [18,19]. The transport protocol uses UDP (User Datagram Protocol) and has the advantage of supporting multicast, but CoAP has a disadvantage in that it is difficult to apply to time critical systems, due to limited QoS policy and asynchronous connection. MQTT-SN (MQTT for Sensor Networks) is a protocol for sensor nodes operating in a process similar to MQTT [20,21]. The benefit is that it can be applied to devices with multicast support and low computing performance. According to the specification, however, MQTT-SN is an additional version of MQTT suitable for wireless sensor networks, and requires additional gateways, such as MQTT-SN gateway, to connect with the MQTT broker [22]. The MQTT-SN-modified MQTT protocol for low power and low bandwidth has a weakness compared to MQTT in large-scale IoT environments due to the changed topic structure and short message size. MQTT is a broker-based pub/sub protocol standardized by OASIS [23]. The MQTT protocol uses a topic to send data. The MQTT protocol, which has the advantage of lightweight middleware, is useful for IoT data exchange, but has problems caused by brokers [24]. The MQTT protocol causes a serious broker queuing delay if large IoT devices transmit data to a single broker. MQTT using brokers solves discovery problems between pub/sub devices, and has the advantage of asynchronous behavior. Broker, however, increases data transmission delays due to data concentration problems at a single point, and is not suitable for use in edge networks where edge nodes are distributed. Park proposed an Emergent-MQTT that uses bandwidth reservation to minimize transmission delays [25]. Emergent-MQTT identifies emergency data and reserves bandwidth, but does not consider data concentration issues. In order to solve the problem of data concentration, Park proposed Direct-MQTT [26]. Direct-MQTT has made a broker-less path to reduce transfer delays. Direct-MQTT using the unicast method has the problem of increasing network congestion when a large number of IoT devices exchange data. In addition, Direct-MQTT does not take into account the QoS levels suggested by the standard MQTT. Banno proposes Interworking Layer of Distributed MQTT brokers (ILDM) to support heterogeneous MQTT brokers in edge networks [27]. An ILDM node is placed between a broker and a client to support data transfer by connecting heterogeneous brokers that reside on multiple edge networks. ILDM nodes are the cause of additional delays and network congestion, due to data exchanged between ILDM nodes.

## 3. Materials and Methods

**Figure 1:**
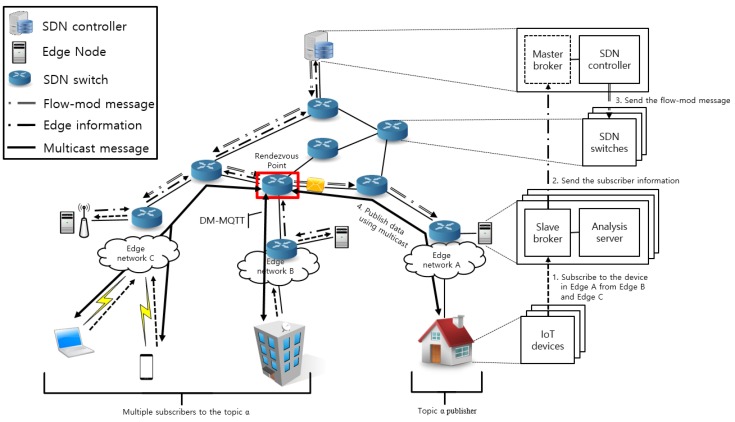
DM-MQTT architecture in edge computing.

The structure of the proposed system is shown in Figure 1. The architecture consists of an SDN controller with MQTT master broker, edge node with MQTT slave broker, and IoT devices.

The MQTT protocol has additional delays due to the centralized structure by which all data is sent to the broker when multiple IoT devices transmit data. Distributing multiple brokers to edge nodes raises complex connectivity issues between IoT devices and brokers across multiple edge networks. Even if the broker-less structure is applied by extending the MQTT protocol, the complexity of the communication structure cannot be solved. In order to solve the above problem, data exchange across the multiple edge networks applies DM-MQTT with broker-less structure and multicast added. Single edge network internal data transmission applies a standard MQTT protocol. The broker-less structure is applied to data transfer between multiple edge networks to reduce the connection complexity of brokers and IoT devices. MQTT protocol applies multicast to reduce the delays that occur during data transmission to multiple subscribers. Group and tree management uses SDN-based multicast instead of IP multicast, which is difficult to manage. Edge information gathered from all edge nodes is passed to the SDN controller through the master broker. The master broker and the SDN controller are logically separated, and the master broker and the SDN controller should be configured on the same server to minimize the data transfer delay and the flow set-up time. The SDN controller manages multicast groups and trees quickly and effectively.

### 3.1. Architecture Overview

**Figure 2:**
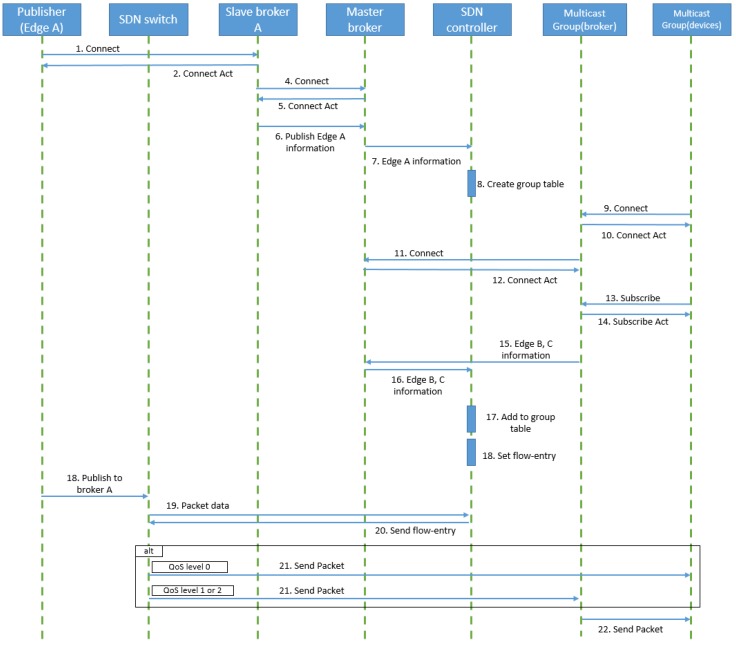
Data transfer sequence diagram of DM-MQTT.

In edge computing, all edge network IoT devices are connected to edge nodes. In order to collect data, all edge nodes have an MQTT slave broker. The analysis server of IoT devices and edge nodes sends and receives data through slave broker. The information of the IoT devices connected to the slave broker is integrated into the edge information and delivered to the master broker. Edge information includes the IP address, topic, QoS level, and emergency data flag of the IoT device. The master broker sends edge information from all slave brokers to the SDN controller. The master broker does not perform the standard MQTT broker function to connect and exchange data with IoT devices. The SDN controller analyzes the edge information, creates a group table, and uses the table to set paths between different edge networks. If multiple subscribers subscribe to the same topic, then a multicast path is configured. The multicast tree type is a core-based tree (CBT). The CBT should set rendezvous point (RP) in the process of establishing the multicast path [28]. The SDN controller measures the delay from the switches to the subscribers. RP is selected to the switch with the smallest average delay. The multicast path made between the IoT device and the RP consists of a bi-directional path. The path generated by the SDN controller is forwarded to the SDN switch, and the data of the particular edge network is transmitted to the other edge network along the set path [29]. The data transfer process into different edge networks is shown in Figure 2.

In Figure 1, it is assumed that the devices belonging to the edge networks B and C subscribe to the data corresponding to the topic p of the edge network A. The IP address of the publisher in edge network A and the topic p are fed into the edge information and forwarded to the SDN controller. The SDN controller analyzes the edge information and creates a group table. The group table contains the publisher IP address, the slave broker IP address connected with the publisher, and the topic. When the devices in edge networks B and C subscribe the topic p to the slave brokers on each edge network B and C, the slave brokers merge the subscriber information into edge information and send it to the master broker. The SDN controller analyzes the edge information of the edge networks B and C transmitted from the master broker. The SDN controller checks the topic p in the group table, writes the device IP address that subscribes to topic p to the table, and sets the path from edge network A to edge network B, C. The final destination address of the data matching to topic p depends on the QoS level set by the subscriber. If the QoS level is 0, data is sent to devices in edge networks B and C. The QoS level is 1 or 2, the destination address is changed to the slave broker in the edge network B, C. Since it is necessary to confirm whether data is received and to retransmit the data, the slave broker connected with the subscriber stores the data. Since the subscriber is in multiple edge networks, the SDN controller creates a multicast group and sends the path to the SDN switch. When an IoT device in edge network A publishes data using topic p, the SDN switch uses the flow entry to transfer data to the destinations in edge networks B and C.

### 3.2. DM-MQTT Operation Process

**Figure 3:**
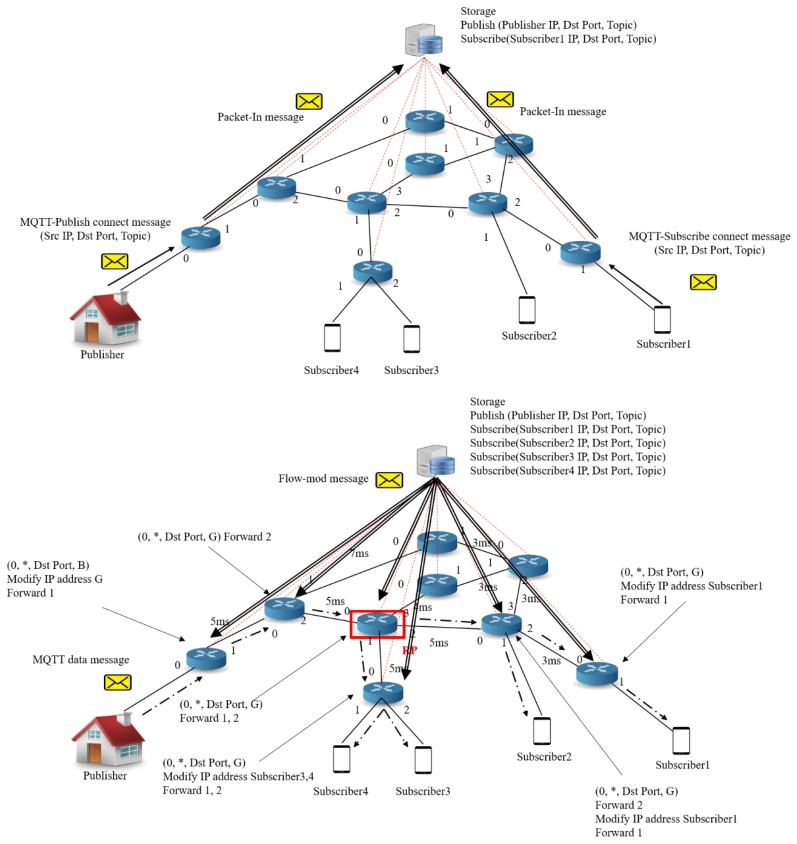
The two types of operation processes in DM-QTT.

The operation process of DM-MQTT is depicted in Figure 3. The DM-MQTT main processes consist of three parts. One part is a multicast group query of MQTT publishers. Another part is a multicast group query of MQTT subscribers. The other part processes construction of a multicast tree which passes MQTT messages. The multicast group query of MQTT publishers operates when the publisher generates to a publish message. The publisher connection messages include parameters in the publisher IP-address, and the topic for the multicast tree group construction. This message transfers to an edge switch. If the edge switch has no a flow entry about the message, the message packs the OpenFlow protocol and forwards to the SDN controller. The SDN controller unpacks the OpenFlow protocol and parses the message for storing publisher information of the multicast tree. The multicast group query of MQTT subscriber acts when the subscriber makes a subscribe message. The subscribe messages contain the subscriber IP address and the topic. The process is similar to the multicast group query process of MQTT publisher, except storing the member information. The construction process of a multicast tree makes a multicast tree in referring to publishers IP address, subscribers IP address, topic, and network link status. DM-MQTT needs to a multicast flow entry in order to implement multicast communication, and requires RP selection algorithm because of the core-based tree(CBT). The DM-MQTT switches are assigned a flow entry of (iff, *, dst_port, G) and (iff, *, dst_port, B). iff means a port in the incoming packet, and * is wildcard which means whole IP address. The parameter of dst_port enables to classify by the port number of MQTT message. G means a multicast group address. B is the broker address. The edge switches install the flow entry of (iff, *, dst_port, B), and have two actions modifying MQTT packet field of an IP destination and forwarding to port for sending MQTT packet from publisher to subscriber. The switches, except for the edge switches, only perform to forward a multicast MQTT packet through (iff, *, dst_port, G) of the flow entry. The RP called to a root of a core-based tree (CBT) is selected through a ratio of pub/sub devices and the measured link delay. DM-MQTT applies to an RP selection algorithm for services about an emergency of many subscribers. The RP selection algorithm chooses the candidate RP having the shortest average delay between candidate RPs and subscribers, based on the measured link delay [30]. DM-MQTT constructs multicast paths connecting between RP and the publisher, or RP and subscriber. The SDN controller sends the flow-mod messages for installing a flow entry referring to made paths. After all the processes are completed, the MQTT message is forwarded from the subscriber to the publisher by the flow of the switch.

### 3.3. Direct Multicast—MQTT

The SDN controller makes a flow entry for data transmission between different edge networks. The following information is required for flow entry.

- Publisher IP address
- The IP address of the slave broker connected with the publisher
- Subscriber IP address
- The IP address of the slave broker connected with the subscriber
- Topic
- Emergency data flag
- QoS level

**Figure 4:**
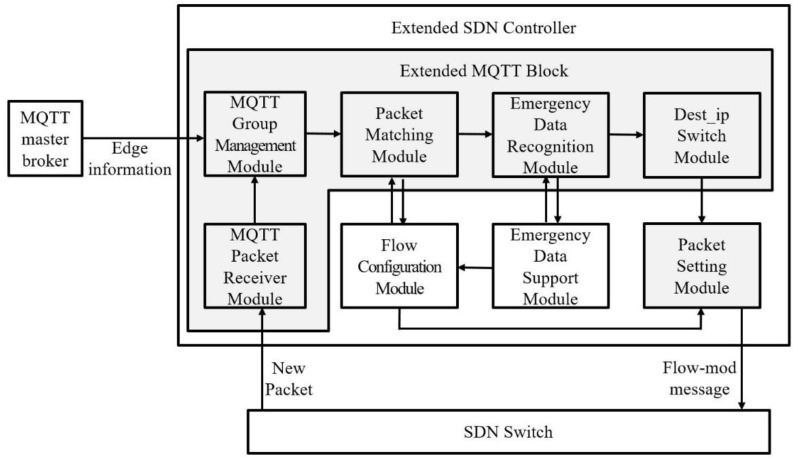
The proposed SDN controller using the extended MQTT block.

The SDN controller sets up groups and generates flow entries through seven pieces of information contained in the edge information sent from the slave broker. The structure of the SDN controller is shown in Figure 4.

When the publisher publishes the data, the SDN switch forwards the MQTT packet to the SDN controller. The MQTT packet receiver module receives the MQTT packet and sends it to the packet matching module together with the group table. The contents of the created group table are classified into subscribers having the same topic as the MQTT packet and transmitted to the flow configuration module. The flow configuration module determines the flow entry based on the topic, the IP address of the pub/sub device, and the QoS level. By default, the flow entry is set to a direct connection between the publisher and the subscriber. If there are multiple subscribers in one topic, a multicast tree is formed. The QoS level of the subscriber is not 0, and the destination address is designated as the slave broker of the edge network belonging to the subscriber. A subscriber whose QoS level is set to 1 or 2 receives data from the slave broker and proceeds with the transmission confirmation and retransmission process. 

Emergency data recognition module identifies emergent-flag in the same way as Emergent-MQTT, and confirms whether it is an emergency message. Emergency data support module reserves bandwidth, to minimize transfer delay in flow entries of packets identified by emergency data. In addition, the emergency data support module detects the packet loss by checking the ports of the switches set in the flow entry. If the emergency data is lost, the flow configuration module generates a new flow entry to ensure stable transmission of the next packet.

**Figure 5:**
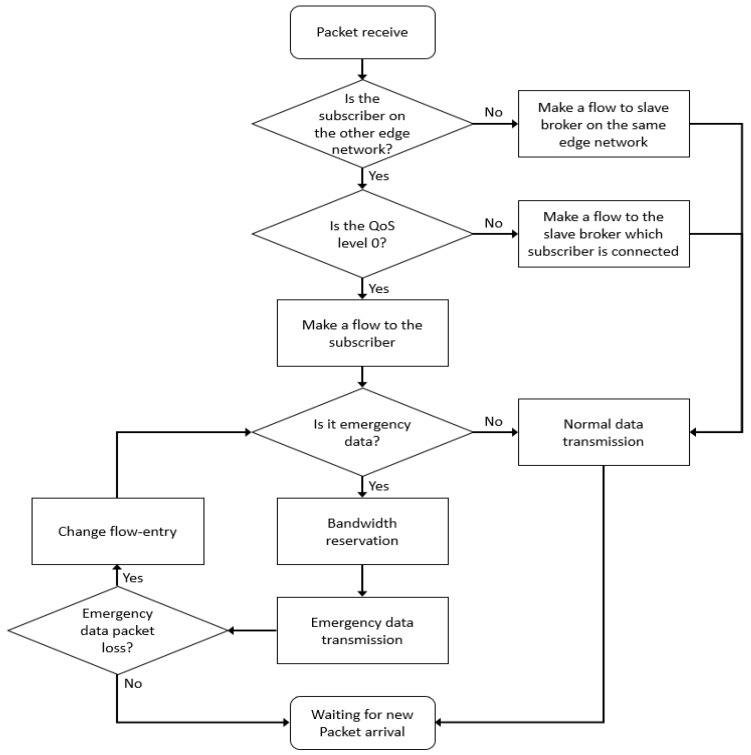
Flowchart of SDN controller routing process.

After the flow-entry configuration and emergency data processing, the dest_ip switch module changes the destination address of the packet and sends a flow-mod message to all SDN switches included in the flow entry in the packet setting module. The SDN switch uses the flow-mode message to transfer the MQTT packet to the changed destination address. Flow-entry configuration and emergency data management in the SDN controller are illustrated in Figure 5.

**Figure 6:**
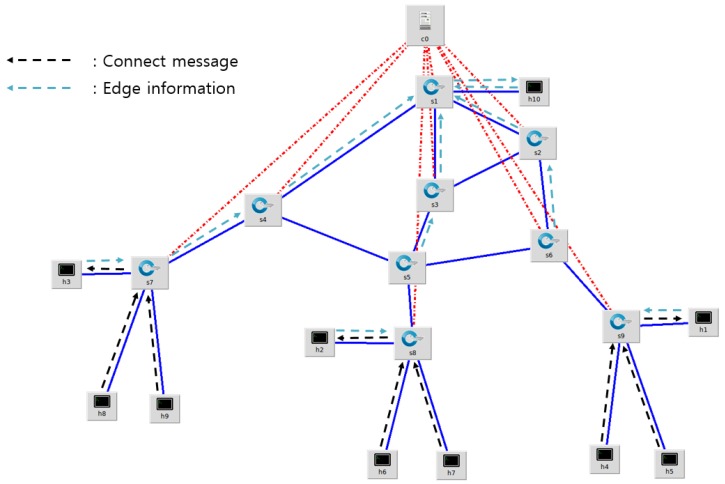
Flow of edge information and connection message path of DM-MQTT in edge networks.

Multiple slave brokers in a multi-edge network send edge information to the master broker. The SDN controller receives all edge information from the master broker. The flow of edge information from each slave broker to the master broker is shown in Figure 6.

In Figure 6, h1, h2, and h3 are edge nodes with slave brokers, and h4, h5, h6, h7, h8, and h9 are considered gateways containing multiple IoT devices. c0 is an SDN controller that connects to all OpenFlow switches to set up flow entries. The master broker h10 collects all edge information and sends it to the SDN controller. In order to run the master broker on the Mininet, the host 10 is separately configured, and it appears as if the SDN controller and the master broker are physically separated in the Mininet editor. However, in the code, the SDN controller and the master broker are logically separated and physically installed on the same hardware, delivering edge information through IPC (Inter-Process Communication). First, IoT devices connected to the edge network transmit a connection message to the slave broker configured on the edge node. The slave broker combines the IP addresses, topics, and QoS levels of the publishers and subscribers configured in the edge network with edge information, and the master broker receives it from the slave broker. The master broker transfers all edge information to the SDN controller. The SDN controller analyzes the edge information using the MQTT group management module of the extended MQTT block and generates the group table shown in Table 2.

Since the MQTT protocol determines whether to transmit data through the same topic, the SDN controller sets up a flow entry after ensuring that the publisher and subscriber topics are identical. In the group table, the subscriber’s IP address is used as the destination address when the SDN controller determines the flow entry of the MQTT packet. If the QoS level of the subscriber is not 0, the Sub_slave broker IP address is used as the destination address. Multicast group column is used when there are multiple subscribers on the same topic as in Table 2. When a multicast subscriber group with the same subject is configured, the flow entry is set to a multicast route.

**Figure 7:**
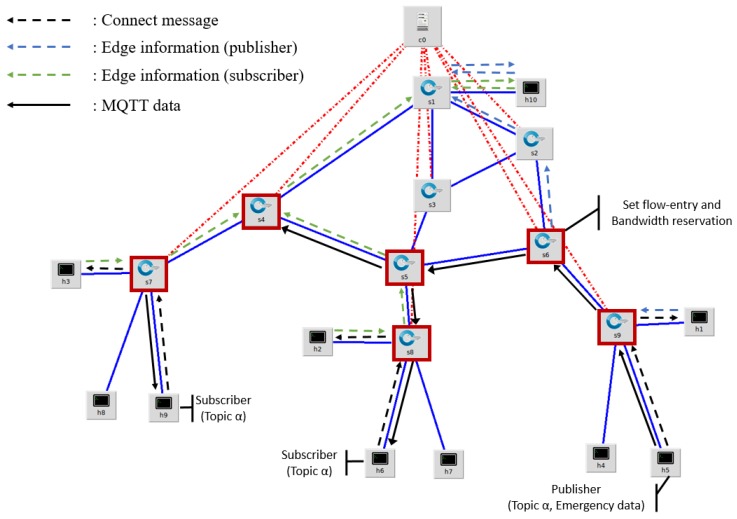
Example of message path of DM-MQTT in edge network.

As a result, the DM-MQTT supports a data transport mechanism that does not require a connection between any broker and all pub/sub devices in multiple edge networks. This mechanism can significantly reduce network congestion, along with setting up multicast routes when multiple subscribers are present. Bandwidth reservation and flow-entry change mechanisms ensure the transmission of emergency data. In addition, DM-MQTT can be compatible with the standard MQTT protocol because it classifies the destination address according to the QoS level without setting the transmission path of all MQTT packets as a broker-less route. The process of subscribing to data on heterogeneous edge networks is shown in Figure 7. The following describes the data transmission order to the external edge network shown in the Figure 7.

1. Subscribers in h6 and h9 forward connection messages that subscribe topic alpha to slave brokers in their existing edge network
2. Slave brokers h2 and h3 send edge information, including subscription information, to master broker h10
3. The master broker sends subscriber edge information to the SDN controller C0
4. The publisher in h5 sets topic alpha as an emergency message and sends a connection message to slave broker h1
5. The slave broker h1 sends the edge information including the topic alpha publications to the master broker
6. The master broker sends publisher edge information to the SDN controller
7. The SDN controller creates a multicast group to make a flow entry and sends a flow-mode message to the S4, S5, S6, S7, S8, and S9 switches
8. The SDN controller sends a bandwidth reservation on the topic alpha to switches
9. Data published from h5 is sent to h6 and h9 according to flow entry

## 4. Results and Discussion

The authors in this paper verify the DM-MQTT by measuring the data transmission delay and the network usage in the data transmission process between different edge networks. The experimental results are compared with the standard MQTT and D-MQTT, the DM-MQTT (QoS = 0), and the DM-MQTT (QoS = 1).

### 4.1. The Experimetal Environments

The network testbed is based on the Mininet emulator [31]. The SDN controller uses the Ryu controller to manage the entire network flow [32]. The topology used in the experiment is the same as in Figure 4. Figure 4 shows three edge networks, each edge network connected to the SDN network via the OpenFlow switches S7, S8, and S9 [33], and edge networks 1, 2, and 3 in accordance with hosts 1, 2, and 3 serving as edge nodes. Hosts 4, 5, 6, 7, 8, and 9, serving as gateways, contain eight publishers and two subscribers, respectively. The master broker and the slave broker used the mosquitto broker. Each host is connected with a slave broker configured on the same edge network. A slave broker is used when data is exchanged inside the edge network. Even if DM-MQTT is used, when the subscriber’s QoS level is 1 or 2, the data is transmitted to the slave broker in the edge network with the subscriber, and executes the retransmission process.

Every publisher publishes a single topic, and each gateway sends a message consisting of eight topics to the slave broker. Four of the 16 topics created in one edge network are sent to the other edge network. Subscribers in edge network 1 subscribe to 16 topics within the edge network, 4 topics from edge network 2, and 4 topics from edge network 3. For example, subscribers in h4 subscribe to 16 topics sent by publishers in h3 and h4, and 8 topics sent by publishers in h6, h7, h8, and h9. Two of the 8 topics sent by publishers in h4 will be delivered to h6, h7, h8, and h9 subscribers. The experiments measure data transfer delays and network usage.

### 4.2. The Data Transfer Delay Performance of DM-MQTT

**Figure 8:**
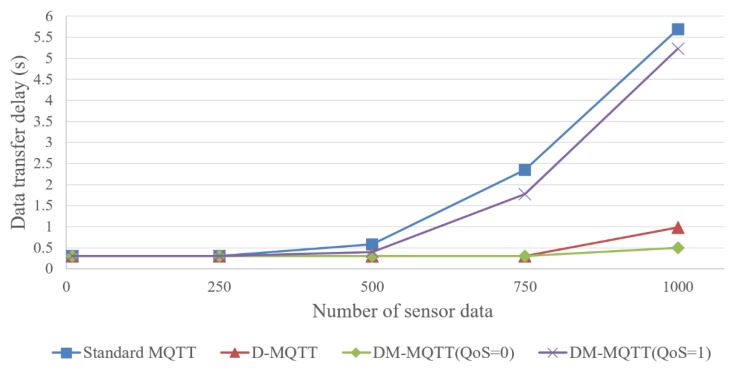
Changes in data transfer delay depending on the number of data samples.

The data transmission delay between different edge networks is shown in Figure 8. The experiment measures the transfer delay while increasing the transmission data. Each publisher increased the number of transmitted data by 250 per experiment. Finally, an experiment was conducted to transfer 1000 data per second. The situation of increasing the amount of data sent means that more publishers are sending the same topic. The transfer delay measures the exchange of data between multiple edge networks.

In the experimental procedure, propagation delay, transmission delay, and queuing delay are set the same in all MQTT protocols. The standard MQTT shows the highest transfer delay and the DM-MQTT (QoS = 1) measures the second highest transfer delay. Since the standard MQTT and DM-MQTT (QoS = 1) send data using a broker, the subscriber must be connected to the broker where the topic is collected. To subscribe to a topic generated in h4, the subscriber of h6, h7, h8, and h9 must be connected to the slave broker built in h1. The broker base mechanism connects at least 26 IoT devices to the slave broker: 16 publishers and 10 subscribers. Whenever there is one publisher for each topic, the queuing delay of the slave broker rarely occurs. If the publisher for each topic increases by more than 500, the queuing delay of the slave broker increases dramatically due to multiple publishers. The DM-MQTT (QoS = 1) using multicast reduces the broker queuing delay slightly compared to the standard MQTT that transmits data to each subscriber. The queuing delay, however, is still serious, and shows that the broker-based mechanism is not suitable for sending emergency data. The DM-MQTT (QoS = 1) using the broker can guarantee the reliability of emergency data, but does not satisfy the promptness.

The D-MQTT and the DM-MQTT (QoS = 0) send data without using a broker, and the transfer delay is lower than that of the DM-MQTT (QoS = 1) because no queuing delay occurs in the broker. Each slave broker is connected to the IoT device of the edge network to which it belongs, and generates a queuing delay in the internal data exchange. The slave brokers, however, are not used in data exchange between multiple edge networks. In D-MQTT and DM-MQTT (QoS = 0) with no delay elements other than propagation delay, transmission delay, and queuing delay, which is fixedly set in the Mininet, a transfer delay close to zero is measured. If the network is highly congested, DM-MQTT (QoS = 0) using multicast may show a lower transfer delay than D-MQTT using unicast. Although the results of DM-MQTT (QoS = 0) and D-MQTT show a similar transfer delay because the experimental environment does not assume network congestion in extreme situations, in experiments using 1000 data samples, the D-MQTT measures slightly higher transfer delays. The systems that process emergency data are time critical systems, and the rapid transfer of data is an important factor. The results in Figure 8 prove that the broker-less mechanism effectively supports promptness.

### 4.3. The Network Usage of DM-MQTT

**Figure 9:**
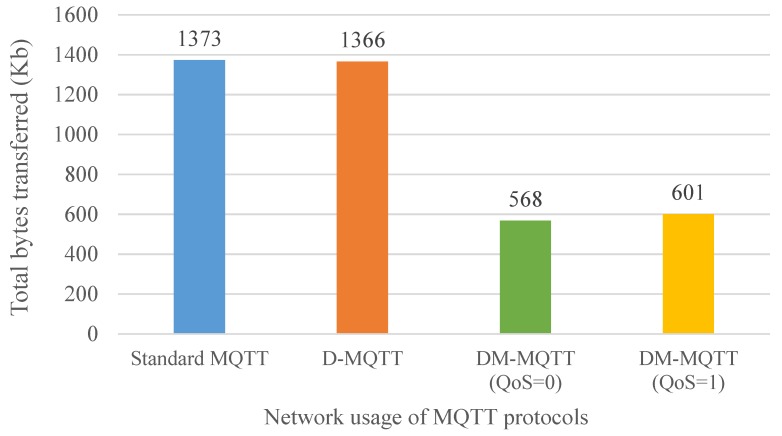
The total number of bytes transferred for each MQTT protocol.

The measurement results of the network usage are shown in Figure 9. The experiment measured the total bytes of packets processed by switch 4 after 5 min of data transmission on all publishers. The packets processed by the switch 4 include both data transmitted from the edge networks 1 and 2 to edge network 3 and data sent from edge network 3 to edge networks 1 and 2.

The total bytes transferred in the standard MQTT and D-MQTT are similar and higher than in DM-MQTT. The standard MQTT and D-MQTT transfer data use unicast. The difference between the standard MQTT and the D-MQTT is whether to send a connection message between the broker and the IoT device. In experiments, when a publisher sends to another edge network, multiple subscribers receive the data. Four publishers belonging to edge networks 1 and 2 forward data with unicast to two subscribers of edge network 3. Two publishers in edge network 3 transfer data unicast to four subscribers in the other edge network. Since every publisher sends two topics to each subscriber, the unicast method has a large number of packets to process. In the experiment, the edge network 3 sends 16 topic data to edge networks 1 and 2, and the incoming data to edge network 3 is also 16 topic data. In switch 4, a total of 32 topic data are exchanged, and the standard MQTT has additional broker connection messages. 

DM-MQTT, however, uses a multicast method regardless of QoS level, and the total number of bytes is smaller than that of standard MQTT and D-MQTT. DM-MQTT uses multicast packet when transmitting data from edge network 3 to edge network 1, 2, even though there are many subscribers. In addition, edge networks 1 and 2 also use multicast packets. With DM-MQTT, switch 4 sends and receives a total of 12 topic data. As a result of the data that is generated by the DM-MQTT to construct a multicast tree, the total number of bytes in the packets processed by switch 4 is greater than the number of bytes in the topic data to be processed. In addition, DM-MQTT has the edge information that is sent from the slave broker to the master broker. The edge information is used to transfer information from IoT devices and slave brokers. In the experiment, IoT devices and brokers do not need to be moved, added, or deleted after the initial assignment, so there is no need to send additional edge information after sending the first edge information once. Experimental results show that DM-MQTT is 58% less than unicast method, even if messages for multicast configuration are added. Even in a simple data transfer experiment using 12 IoT devices, the number of bytes processed by the switch is effectively reduced. In massive edge network situations, where many IoT devices exist in multiple edge networks and exchange data with each other, the effect of reducing the network congestion of DM-MQTT by applying multicast is expected to increase even more. Reduced network congestion can efficiently use fixed network bandwidth with less chance of additional data transfer delays due to congestion. In conclusion, congestion reduction affects the reduction of data transfer delay.

## 5. Conclusions

The MQTT protocol requires a broker distribution in accordance with an edge computing paradigm suitable for large-scale systems with multiple IoT devices. The MQTT protocol, where data is collected and transmitted through a broker, is useful for small-sized edge networks by distributed brokers, but additional problems, such as increased network congestion and data transmission delays, can occur during data transmission between different edge networks. The authors in this paper propose DM-MQTT with hierarchical broker structure to solve these problems. DM-MQTT builds a slave broker on the edge node of all edge networks, and the master broker gathers edge information from the slave broker. The SDN controller uses the edge information received from the master broker to set the data transmission path between different edge networks. This mechanism reduces network usage by allowing data to be transferred without connections between brokers and devices on different edge networks. In addition, when there are multiple subscribers, multicast is used to effectively reduce data transfer delays and network usage. The SDN-based multicast mechanism using edge information enable smooth operation of DM-MQTT multicast by easily managing multicast groups and trees compared to IP multicast. As a result, DM-MQTT reduced data transfer delays by 65% and reduced network usage by 58%, compared to standard MQTT. We believe that DM-MQTT can reduce the congestion of networks in large IoT environments and quickly transfer critical data to other edge networks if disasters and accidents cannot be handled in a single edge network. DM-MQTT does not take into account the mobility of IoT devices. As the mobility of IoT devices increases, edge information updates caused by joining and leaving edge networks are required. If an IoT device is added to the edge network and connected to the slave broker on the edge node or deleted on the edge network, the slave broker must update the edge information and send it to the master broker. If IoT devices exhibit dynamic mobility, then you should consider how to manage increasing edge information and quickly reconfigure multicast trees.

## Figures and Tables

**Table 1:** Functional Comparisons of IoT Middleware.

	MQTT	MQTT-SN	CoAP	DDS
Data transmission	Broker Pub/Sub	Broker Pub/Sub	RESTful	Pub/Sub
QoS	3 policies	3 policies	Partial	22 policies
Real-time	Non-real time	Non-real time	Non-real time	Soft real time
Multicast support	Non	Support	Support	Support
Transport Protocol	TCP (Transmission Control Protocol)	UDP	UDP	UDP

**Table 2:** Group table created by MQTT group management module.

Publisher IP	Pub_slave Broker IP	Subscriber IP	Sub_slave Broker IP	Topic	QoS Level	Multicast Group
10.0.0.1	10.0.0.2	-	-	temperature	0	
-	-	10.0.0.5	10.0.0.6	temperature	0	A
-	-	10.0.0.10	10.0.0.11	temperature	0	A

## References

[1] Evans D. (2011). The Internet of Things: How the next evolution of the Internet is changing everything. CISCO White Pap..

[2] Greenberg A., Hamilton J., Maltz D.A., Patel P. (2008). The cost of a cloud: Research problems in data center networks. ACM SIGCOMM Comput. Commun. Rev..

[3] Taleb T., Dutta S., Ksentini A., Iqbal M., Flinck H. (2017). Mobile edge computing potential in making cities smarter. IEEE Commun. Mag..

[4] Self-Driving Cars Will Create 2 Petabytes of Data, What Are the Big Data Opportunities for the Car Industry. http://www.computerworlduk.com/news/data/boeing-787screate-half-terabyte-of-data-per-flight-says-virgin-atlantic-3433595/.

[5] Data Never Sleeps 2.0. https://www.domo.com/blog/2014/04/data-never-sleeps-2-0/.

[6] Popa L., Kumar G., Chowdhury M., Krishnamurthy A., Ratnasamy S., Stoica I. (2012). FairCloud: Sharing the network in cloud computing. ACM SIGCOMM Comput. Commun. Rev..

[7] Rahmani A.M., Gia T.N., Negash B., Anzanpour A., Azimi I., Jiang M., Liljeberg P. (2018). Exploiting smart e-Health gateways at the edge of healthcare Internet-of-Things: A fog computing approach. Futur. Gener. Comput. Syst..

[8] Roca D., Milito R., Nemirovsky M., Valero M. (2018). Tackling IoT Ultra Large Scale Systems: Fog computing in support of hierarchical emergent behaviors. Fog Computing in the Internet of Things.

[9] Aazam M., Huh E.N. Fog computing micro datacenter based dynamic resource estimation and pricing model for IoT. Proceedings of the 2015 IEEE 29th International Conference on Advanced Information Networking and Applications.

[10] Weisong S., Cao J., Zhang Q., Li Y., Xu L. (2016). Edge computing: Vision and challenges. IEEE Internet Things J..

[11] Xiang S., Ansari N. (2016). EdgeIoT: Mobile edge computing for the Internet of Things. IEEE Commun. Mag..

[12] Al-Fuqaha A., Guizani M. (2015). Internet of things: A survey on enabling technologies, protocols, and applications. IEEE Commun. Surv. Tutor..

[13] Govindan K., Azad A.P. (2015). End-to-end service assurance in IoT MQTT-SN. Proceedings of the 2015 12th Annual IEEE Consumer Communications and Networking Conference (CCNC).

[14] Roy D.G., Mahato B., De D., Buyya R. (2018). Application-aware end-to-end delay and message loss estimation in Internet of Things (IoT)—MQTT-SN protocols. Future Gener. Comput. Syst..

[15] McKeown N. Software-defined networking. Proceedings of the INFOCOM Keynote Talk.

[16] Bertaux L., Hakiri A., Medjiah S., Berthou P., Abdellatif S. (2014). A DDS/SDN based communication system for efficient support of dynamic distributed real-time applications. Proceedings of the 2014 IEEE/ACM 18th International Symposium on Distributed Simulation and Real Time Application.

[17] Choi H.Y., King A.L., Lee I. (2016). Making DDS really real-time with OpenFlow. Proceedings of the 13th International Conference on Embedded Software.

[18] Huh J.H., Kim D.H., Kim J.D. (2016). oneM2M: Extension of protocol binding: Reuse of binding protocol’s legacy services. Proceedings of the 2016 International Conference on Information Networking (ICOIN).

[19] Bormann C., Castellani A.P., Shelby Z. (2012). Coap: An application protocol for billions of tiny internet nodes. IEEE Internet Comput..

[20] Schütz B., Bauer J., Aschenbruck N. (2016). Improving Energy Efficiency of MQTT-SN in Lossy Environments Using Seed-Based Network Coding. Proceedings of the 2017 IEEE 42nd Conference on Local Computer Networks (LCN).

[21] Amaran M.H., Noh N.A.M., Rohmad M.S., Hashim H. (2015). A comparison of lightweight communication protocols in robotic applications. Procedia Comput. Sci..

[22] Stanford-Clark A., Truong H.L. (2013). MQTT for Sensor Networks (MQTT-SN) Protocol Specification.

[23] Kim J., Choi S.C., Yun J., Lee J.W. (2018). Towards the oneM2M Standards for Building IoT Ecosystem: Analysis, Implementation and Lessons. Peer-to-Peer Networking and Applications.

[24] Xu Y., Mahendran V., Radhakrishnan S. (2016). Towards SDN-based fog computing: MQTT broker virtualization for effective and reliable delivery. Proceedings of the 2016 8th Communication Systems and Networks (COMSNETS).

[25] Park J.H., Yun S., Kim H., Kim W.T. (2017). Emergent-MQTT over SDN. Proceedings of the 2017 International Conference on Information and Communication Technology Convergence (ICTC).

[26] Park J.H. Dependable fire detection system with multifunctional artificial intelligence framework. IEEE Netw..

[27] Banno R., Sun J., Fujita M., Takeuchi S., Shudo K. (2017). Dissemination of edge-heavy data on heterogeneous MQTT brokers. Proceedings of the 2017 IEEE 6th International Conference Cloud Networking (CloudNet).

[28] Lin Y.-D., Lai Y.-C., Teng H.Y.Y., Liao C.C.C., Kao Y.C. (2017). Scalable multicasting with multiple shared trees in software defined networking. J. Netw. Comput. Appl..

[29] Braun W., Menth M. (2014). Software-defined networking using openflow: Protocols, applications and architectural design choices. Future Internet.

[30] Kim H., Yun S., Kim H., Kim W.T. (2017). A novel SDN multicast for large-scale IoT environments. Proceedings of the 2017 International Conference on Information and Communication Technology Convergence (ICTC).

[31] De Oilveira R.L.S., Shinoda A.A.A., Schweitzer C.M.M., Prete L.R. (2014). Using Mininet for emulation and prototyping Software-Defined Networks. Proceedings of the 2014 IEEE Colombian Conference on Communications and Computing (COLCOM).

[32] Oktian Y.E., Lee S.G., Lee H.J. (2017). Distributed SDN controller system: A survey on design choice. Comput. Netw..

[33] (2015). OpenFlow Switch Specification.

